# Supplementary material for: Parallel motion vision pathways in the brain of a tropical bee
Source: J Comp Physiol A Neuroethol Sens Neural Behav Physiol. 2023 Apr 5;209(4):563–91. doi: 10.1007/s00359-023-01625-x (PMC10354172; doi:10.1007/s00359-023-01625-x)
Supplement: Supplementary file 1 — Supplementary file1 (PDF 4975 KB) [file 359_2023_1625_MOESM1_ESM.pdf]

Supplementary Figures

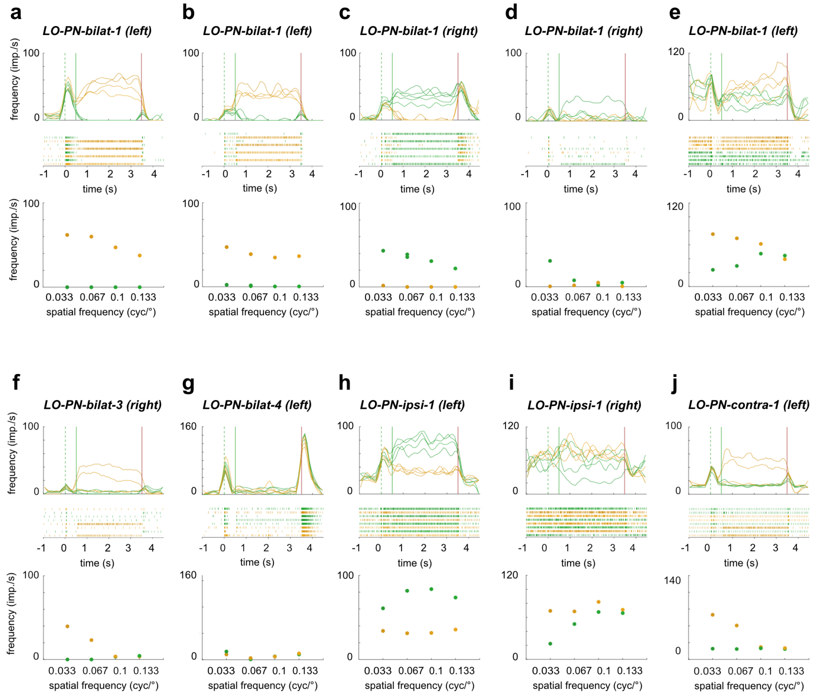

Supplementary Figure 1. Spatial frequency tuning of lobula projection neurons (LO-PNs). (a-j) Top: Activity of different types of LO-PNs in response to clockwise (yellow) and counter-clockwise (green) wide-field optic flow of different spatial frequencies (for spatial frequency values see bottom graph). Green dotted lines: grating presented; green solid lines: motion onset; red lines: motion stop. Middle: Corresponding raster plots. Bottom: Mean frequency during the final 2 s of each stimulus bout. No ND filter in (a-c,e,h).

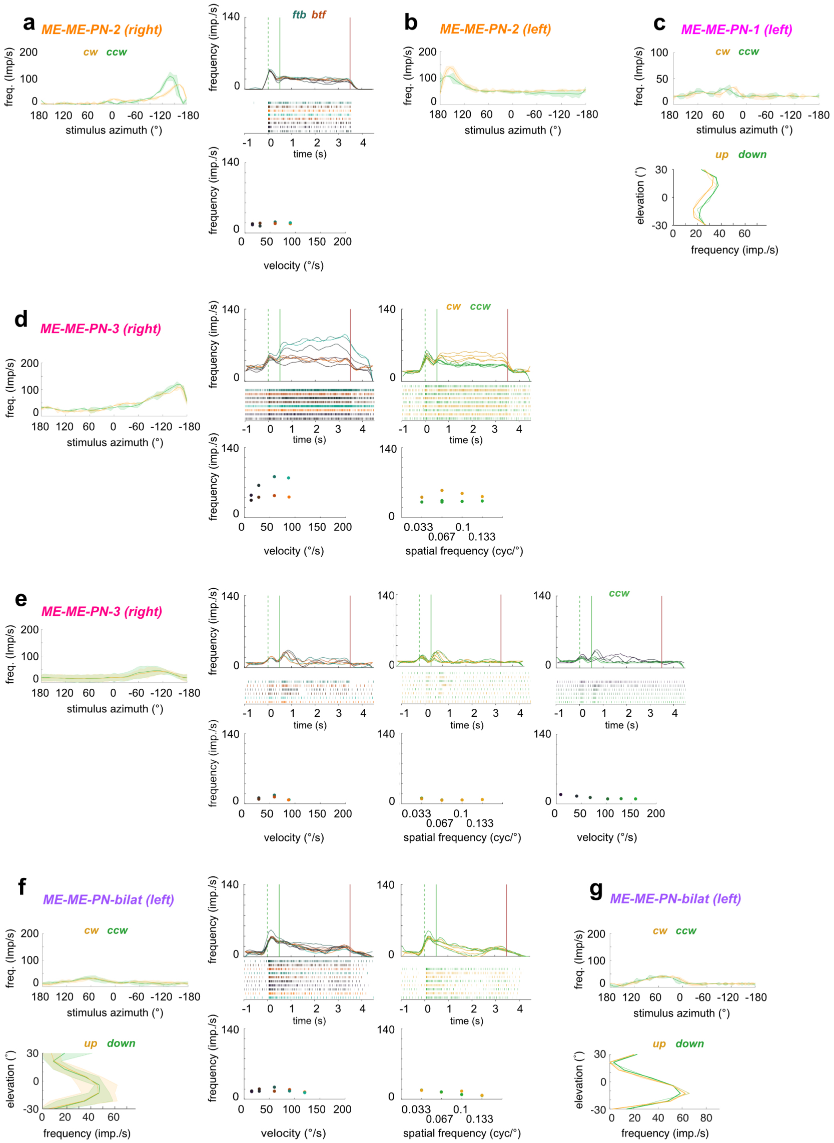

Supplementary Figure 2. Physiological data for all ME-ME-PN-1,-2,-3, and -bilat neurons. Horizontal receptive fields of all cells (a,d,e: left; b; c,g: top; f: top left corner) were mapped with a green vertical bar moving around the animal either clockwise (cw, yellow) or counter-clockwise (ccw, green). Vertical receptive fields of three cells (c,g, bottom; f, bottom left corner) were mapped with a horizontal bar moving up (yellow) or down (green). Activity in response to front-to-back (ftb) or back-to-front (btf) optic flow of different velocities was recorded in one ME-ME-PN-2 (a, right graphs), both ME-ME-PN-3 (d,e) and one ME-ME-PN-bilat (f). Activity in response to cw and ccw optic flow of different spatial frequencies was recorded in both ME-ME-PN-3 cells (d,e) and in one ME-ME-PN-bilat cell (f). Activity in response to ccw optic flow of different velocities was recorded in one ME-ME-PN-3 cell (e, graphs on the right). No ND filter in (d,e), one ND filter in (g).

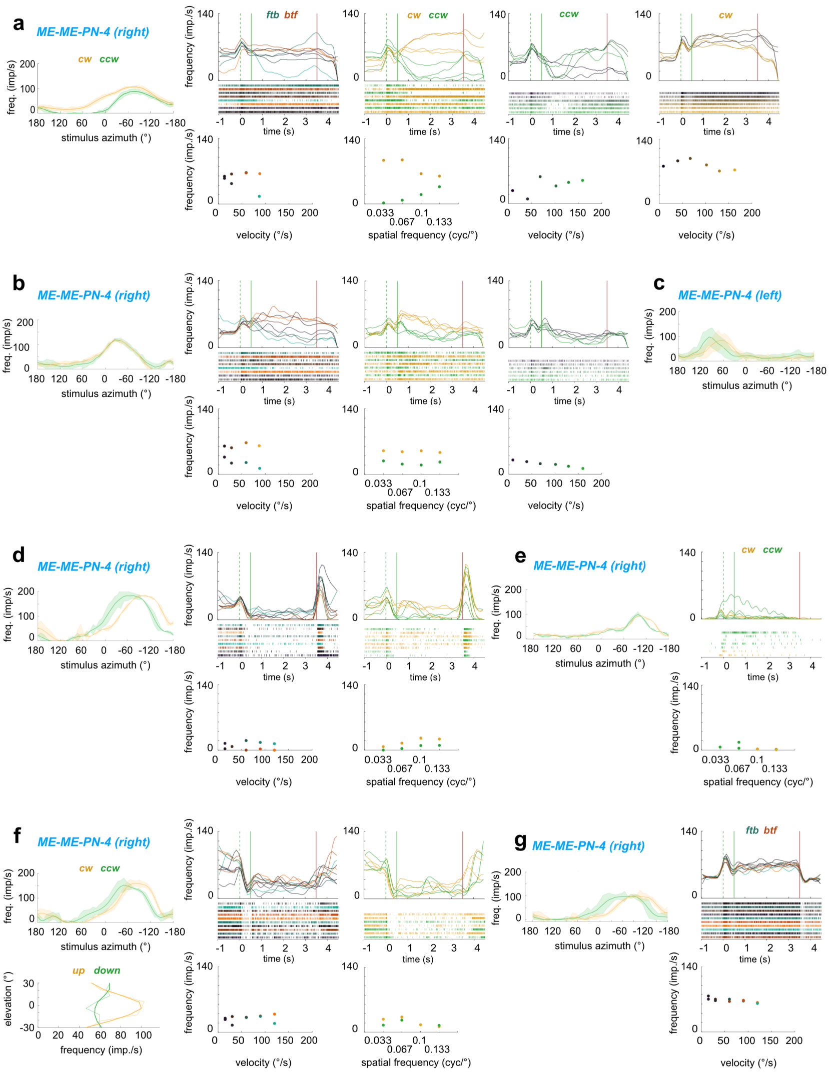

Supplementary Figure 3. Physiological data for all ME-ME-PN-4 neurons. Horizontal receptive fields of all cells (a,b,d-g: left; c) were mapped with a green vertical bar moving around the animal either clockwise (cw, yellow) or counter-clockwise (ccw, green). Vertical receptive fields of one cell (f, bottom left corner) was mapped with a horizontal bar moving up (yellow) or down (green). Activity in response to front-to-back (ftb) or back-to-front (btf) optic flow of different velocities was recorded in five out of seven cells (a,b,d,f,g). Activity in response to cw and ccw optic flow of different spatial frequencies was recorded four cells (a, b, d, f). Activity in response to cw and ccw optic flow of different velocities was recorded in one cell (a). Activity in response to ccw optic flow of different velocities was recorder in one cell (b). No ND filter in (a,b,d,e).
